# Structure-Activity of Plant Growth Bioregulators and Their Effects on Mammals

**DOI:** 10.3390/molecules29235671

**Published:** 2024-11-29

**Authors:** Zeno Garban, Gheorghe Ilia

**Affiliations:** 1 Biochemistry and Molecular Biology, University of Life Sciences “King Michael I”, 119 Aradului Ave., 300645 Timisoara, Romania; zeno.garban@yahoo.com; 2 Working Group for Xenobiochemistry, Romanian Academy—Timisoara Branch, 24 M. Viteazu Ave., 300223 Timisoara, Romania; 3 Department of Biology-Chemistry, West University Timisoara, 16 Pestalozzi Str., 300223 Timisoara, Romania

**Keywords:** plant growth bioregulators, activity, effects, plants, mammals

## Abstract

In this review, we emphasize structure-activity and the effects on mammals of plant growth bioregulators. plant growth bioregulators can be referred to as “biochemical effectors” since they are substances having biological activity. It is possible to distinguish between “bioregulators” and “regulators” due to the significance of the compounds mentioned above in biochemistry and agrobiology. Thus, “plant growth bioregulators” (PGBRs) are the names given to naturally occurring chemical substances produced by biosynthetic processes. PGBRs affect both plant reign and animal reign. A plethora of plant growth bioregulators were described in the literature, so the structure, activity in plants, and their effects on mammals are presented.

## 1. Introduction

Plants need light, water, oxygen, minerals, and other nutrients to grow and flourish. In addition to these environmental requirements, plants rely on certain chemical substances to signal, regulate, and control their growth. These are collectively known in the literature as plant growth (bio)regulators or plant growth hormones. In the late 19th century, a series of observations and tests on geotropism and heliotropism led to the discovery of chemicals acting as chemical messengers among plant tissues [1]. Plant growth (bio)regulators (PG(B)Rs) are chemicals that, when administered to plants in low concentrations, can increase yields, modify growth patterns, enhance nutritional quality, and provide resistance to different stressors (cold, heat, drought, insect, disease, and salt). PG(B)Rs are known to enhance the source-sink relationship and accelerate photosynthesis, minimizing flower drop, encouraging fruit and seed development, and finally enhancing crop yield [2]. PG(B)Rs are endogenous or synthetically created chemicals that may govern certain biochemical and physiological activities in numerous species, most likely through gene and enzyme interactions [3]. Plant tissues create PGBRs spontaneously (endogenously), which enhances their growth and development. These chemicals, which are normally active at very low levels, work by influencing, altering, or regulating plant growth processes, such as the creation of leaves and flowers, stem elongation, fruit development, ripening, and so on [4]. PGBRs are chemicals with biological action, which is why they are known as “biochemical effectors”. Plant growth regulator (PGR) is the general term used in chemistry and biology to refer to certain organic chemical substances that govern physiological processes peculiar to plant species. Given the importance of the aforementioned chemicals in biochemistry and agrobiology, a distinction can be made between “bioregulators” and “regulators”. As a result, natural chemical compounds derived from biosynthetic processes will be known as “plant growth bioregulators” (PGBRs). In addition to this nomenclature, chemical molecules produced by organic chemical synthesis will be referred to as “plant growth regulators” (PGRs). They are used in plant physiology, biochemistry, and molecular biology research. It applies to certain agricultural technology and has practical applications. In low doses, these compounds influence the processes of growth, differentiation, and development known as morphogenesis (for example, germination, sprouting, blooming, fertilization, and ripening). PGBRs are biosynthesized in meristematic tissue; there is no glandular system similar to that of animals. Initially, it was considered that these compounds lacked protein receptors. Currently, it is understood that specific protein receptors serve as bioregulators or regulators [5]. Having the attribute of biologically active substances, bioregulators/growth regulators in plants have aroused interest in biochemistry and cell biology for the reason that they participate in biochemical interactions specific to metabolic processes. Logically, it is necessary to distinguish between “plant growth bioregulators” (PGBRs)—for compounds formed by biosynthesis in plant cells—and “plant growth regulators” (PGRs)—for compounds synthesized in the laboratory. The name “plant hormones” (phytohormones) is also found in various scientific works. The name “hormones” used for signal molecules with an endocrine role in higher animals and humans was introduced by Bayliss and Starling [6]. There is the idea that, similar to hormones, in the case of PGBRs and PGRs, they have acceleration/inhibition effects on plant physiology and there is a transit of plant tissues. The physiological role of hormones in higher animals and humans differs a lot in that, in their case, there is general coordination at the pituitary level and interrelationships between various hormones (e.g., thyroid, pancreatic, and gonadal), which are dependent on the pituitary and nervous system (central and vegetative). In treatises on animal and human physiology, such problems are explained in detail considering hormonal interrelationships. Considering the observations of physiologists briefly explained below, it is normal to use appropriate and unmistakable terminology. So, the designations of PGBRs will be used for biosynthesis compounds present in plants: PGRs for compounds obtained by synthesis and administered in agricultural/horticultural practice. This paper will present the problem of plant growth bioregulators, referring to their main characteristics: (a) they are endogenous substances—so they are synthesized in the body of plants; (b) show activity in small doses (on the order of mM); and (c) considered information vectors that reach target cells. The abovementioned points distinguish between substances with the role of biologically active substances, in the case of PGBRs, and trophic substances specific to the vegetable kingdom. PGBRs affect both plant reign and animal reign. Due the number of papers related to the term PGRs, in this review, we emphasize the importance of PGBrs for plant and mammal (human) health.

## 2. Plant Growth Bioregulators

Plant scientists often focus on five plant hormones, auxins, cytokinins, ethylene, gibberellins, and abscisic acid, which play a crucial role in influencing many processes, but new bioregulators are being added to the list. A plethora of plant growth bioregulators are described in the literature and a short description of them is presented below (in alphabetical order).

### 2.1. Abscisic Acid

Abscisic acid (ABA) is a “classical” growth inhibitor found *Acer pseudoplatanus* buds by Eagles and Wareing in 1963, and named dormin [7]. In the same era, Addicott identified another chemical, abscisin-II, a substance that controls the abscission of cotton fruits. Both compounds are often named abscisic acid.

The stems, leaves, fruits, and seeds of plants produce this growth inhibitor. Abscisic acid primarily acts as an antagonist to gibberellic acid. It is also known as the stress hormone since it helps plants tolerate various sorts of stress [8].

### 2.2. Auxins

One of the most essential plant hormones is auxin, which promotes plant development. The term “Auxin” comes from the Greek word for “to grow”. These chemicals were the first major plant growth regulators identified. In the 1920s, the Dutch scientist Frits Warmolt Went [9] defined auxins and their role in plant development for the first time. Kenneth V. Thimann [10] isolated and identified the chemical structure of indole-3-acetic acid, the chief of naturally occurring auxins.

Auxins are typically synthesized in stems and roots and then distributed throughout the plant. The most significant auxins are indole-3-acetic acid and indole butyric acid, both derived from natural plant sources. Naturally occurring (endogenous) auxins in plants are also 4-chloroindole-3-acetic acid, phenylacetic acid, and indole-3-propionic acid. Naphthalene acetic acid and 2, 4-dichlorophenoxyacetic acid are obtained through synthetic routes [11].

### 2.3. Brassinosteroids

Brassinosteroids (BRs) are polyhydroxylated sterols found throughout the plant kingdom. They were first discovered in *Brassica napus* pollen.

BRs were also named “brassins”. The most active BR, brassinolide (BL), was discovered in 1979 [12]. The most notable finding was Brassinosteroid-insensitive 1 (BRI1), a receptor kinase that initiates intracellular signaling in response to extracellular BR detection [13]. Other chemically different BRs have been found throughout the plant kingdom, including green algae and land plants, since the discovery of BL, showing that BRs first appeared early in plant evolution. The discovery of BR-deficient mutants in *A. thaliana* resulted in their recognition as plant hormones. Since their discovery in the 1970s, around 70 different naturally occurring BRs have been identified. It is a plant hormone that promotes growth and development. They are quite similar to animal steroid hormones, both structurally and physiologically [14].

### 2.4. Cytokinins

Miller et al. found that adding aged herring sperm deoxyribonucleic acid (DNA) to tobacco stem explants enhanced cell proliferation, which led to the discovery of cytokinins [15]. The addition of fresh herring sperm DNA did not promote cell division. Autoclaving fresh DNA with the medium was shown to increase cell proliferation. The active ingredient, kinetin, was extracted from autoclaved DNA and celebrated for its ability to accelerate cell proliferation. Kinetin T was the first cytokinin found. Cytokines with similar molecular structures, such as 6-(γ, γ-dimethylallyl amino)-purine, 6-benzyl adenine, and zeatin, were also discovered [16]. Cytokinins play a crucial role in plant development, physiology, and environmental response. There are nearly 200 naturally occurring and synthetic cytokinins, including zeatin, a naturally occurring cytokinin [17].

### 2.5. Ethylene

Ethylene, the most used PGBR, is synthesized by plant organs, including ripening fruits and aging tissues.

It is a gaseous plant growth bioregulator. The discovery of the effects of ethylene on plants can be dated back to examinations in the 1800s of the effects of smoke and illuminating gas. The first known case of lighting gas causing plant harm happened in 1858 when George Fahnestock saw illuminating gas leaking from pipes in a greenhouse located in Philadelphia [18]. In 1896, Dimitry Neljubow [19] noticed ethylene as an active component in lighting gases that have an impact on plants. He showed that ethylene changed the development of etiolated pea seedlings, resulting in shorter, thicker epicotyls and diageotropism. This was the first time that ethylene was found to have a biological effect. Since then, a lot of papers have been published relating to this simple and very useful molecule.

### 2.6. Gibberellins

Gibberellins (GAs) are tetracyclic diterpenoid hormones with an ent-gibberellane structure that play an important role in plant growth and development, including the germination of seeds, flowering transition, and fruit morphogenesis [20]. Large-scale fermentation procedures allowed two laboratories in the United States and the United Kingdom to isolate a novel kind of active GA known as gibberellic acid (GA3).

More than 100 varieties of gibberellins, primarily derived from fungi and higher plants, are labeled as GA1, GA2, GA3, and so on, and are members of a large chemical family [21].

### 2.7. Jasmonates

Jasmonates (JAs) are cyclopentanones generated from lipids, including jasmonic acid (JA) and its many derivatives. JAs, as well as free acid, were considered “new” members of PGBRs.

The methyl ester of JA (MeJA) was detected as a dominant smell in the ethereal oil of Jasminum grandiflorum flowers [22]. Jasmonic acid (JA), generated from the fatty acid linolenic acid, was extracted from the culture filtrates of the fungus Botryodiplodia theohromae, and its structure was identified [23]. From higher plants, *Cucurbita pepo* [24], and *Vicia faba* [25], JA was also isolated. Jasmonates have been detected in 150 families and 260 species, including mosses, fungi, and ferns, indicating that they may be widespread in plants. JA is a lipid-derived plant chemical with structural similarities to mammalian prostaglandins [26].

### 2.8. Karrikins

Karrikins, (molecular mass: ~150 Da), a distinct class of plant growth bioregulators, were identified in the smoke of burned plants. In approximately 1200 species from 80 genera, smoke from burning plant debris causes a significant increase in seed germination in fields. Smoke from a variety of biotic sources, including straw, wood, and dry or fresh plant mixtures, may significantly boost germination.

Karrikinolide (the butenolide, 3-methyl-2H-furo [2,3-c]pyran-2-one) was identified in 2003 as a potent germination stimulant found in plant-derived smoke from aqueous extracts from heated plant material and burned wood [27,28,29].

Details on its isolation have been reported [30]. Six karrikins (KAR1–KAR6) were identified from smoke water samples and validated by chemical synthesis, while most biological research has employed KAR1, KAR2, and KAR3.

### 2.9. Melatonin

Melatonin (N-acetyl-5-methoxytryptamine) is a harmless biological substance generated by the pineal gland of animals and many plant tissues. Melatonin may be found in over 20 plant groups, including dicotyledonous and monocotyledonous [31].

Its tremendous impact on plant systems attracted the attention of scientists from several fields in plant sciences [32]. It is a key indoleamine neurotransmitter involved in several radical processes and a significant plant metabolite [33].

### 2.10. Nitric Oxide

Robert F. Furchgott, Louis J. Ignarro, and Ferid Murad received, in 1998, the Nobel Prize in Physiology or Medicine for their discovery of nitric oxide as a signaling molecule. Even though ethylene had been found much earlier, the Nobel Committee said that this was the first discovery of a gas that may operate as a biological signaling molecule. Nitric oxide (NO) is a new and fascinating subject in plant biology. However, because of its chemical features (being a gas, free radical, extremely diffusible, and reactive), the results acquired to date are more curious than exciting. Klepper initially identified NO in plants [34]. Plants use NO to regulate physiological processes, including germination, blooming, and leaf senescence, as well as to respond to environmental challenges [35]. Although much research has shown the many functions and the reactivity of NO in photosynthetic organisms, it remains a problematic topic in the field.

### 2.11. Peptides

Peptides are a novel category of PGBRs with signaling attributes [36,37]. They show significant biological actions at relatively low doses (10^−7^–10^−9^ M). These findings highlight the role of peptides in controlling plant development. More than 30 peptide families were found in plants, with many more identified in plant-interacting organisms, such as bacterial and fungal pathogens, plant-parasitic nematodes, and symbiotic and plant-beneficial bacteria and fungi [38].

### 2.12. Phenolic Compounds

Plants generate phenolic chemicals to promote growth, development, and protection. Phenolic chemicals can attract and repel various organisms in the environment [39].

There are over 8000 phenolic structures, which are classified as phenols and benzoquinones, naphthoquinone, and acetophenone, phenolic, phenylacetic, hydroxycinnamic acids, polypropene, coumarin, isocoumarin, lignans, neolignans, xanthone, stilbene, anthraquinone, flavonoids, isoflavonoids, biflavonoids, lignins, and condensed tannins [40].

### 2.13. Polyamines

Polyamines (PAs) are aliphatic bases with low-molecular-weight-containing amino groups. They are found in nearly all cells generated by organisms during metabolism. PAs are recognized as a distinctive kind of plant bioregulator due to their involvement in a wide range of plant growth and developmental processes, as well as stress responses. There are several types of PAs. Polyamines are present in both eukaryotic and prokaryotic cells in higher plants, as well as in plant RNA viruses and plant tumors [41]. PAs are mostly found in free forms. The primary PAs in plants are putrescine, spermidine, and spermine, which regulate a variety of physiological activities [42].

Other PAs are only found in certain plants or in particular conditions.

### 2.14. Strigolactones

Strigolactones, novel PGBRs, promote parasitic weed seed development, prevent shoot branching, and function as branching factors for AM fungus. Strigolactones (SLs) are a kind of plant hormone that regulates developmental processes and helps plants respond to biotic and abiotic challenges. The first strigolactone, strigol, was identified in cotton root exudate [43], and it took around 40 years for scientists to recognize that SLs are a novel family of phytohormones [44]. Semiochemicals are physiologically active compounds that help spread information across organisms. Witchweed (*Striga* spp., *Orobanchaceae*/*Scrophulariaceae*) and broomrape (*Orobanche* spp., *Orobanchaceae*) are parasitic weeds that rely solely on allelopathic compounds [45].

### 2.15. Turgorins

Ricca found in 1916 that compounds in plant extracts may cause leaf motion in *Mimosa pudica*. Turgorins are a set of signaling molecules that mediate “nastic” motions in plant parts in response to temperature, wounding, mechanical shocks, and other stimuli based on changing turgor pressure.

Although chemicals may be recognized as the foundation of all movement control processes, a wide range of initial inputs can set them into action. Schildknecht discovered 4-O-(6′-O-sulpho)glucoside of gallic acid as the first turgorin responsible for nyctinastic movement in plants. The factors of heat (thermonasty), chemical (chemonasty), touch (thigmonasty), shock (seismonasty), light (photonasty), and so on might all be initial irritants [46,47].

## 3. Biological Activity of Plant Growth Bioregulators

Due to the large number of papers related to the function of PGBRs, it is quite difficult to emphasize all the functions of these compounds. Here we tried to synthesize the most important functions related to the main classes. For more details, see the cited literature.

### 3.1. Abscisic Acid

Abscisic acid is a key molecule that regulates development, growth, and stress responses in plants. Absisic acid has an important function in many physiological processes, such as the maturation and development of seeds; stimulates the closing of the stomata in the epidermis; inhibits plant metabolism; is involved in regulating abscission and dormancy; accelerates leaf abscission in cotton plants; regulates fruit dropping; induces seed dormancy, assists in desiccation and many undesirable growth factors; provides abiotic stress tolerance; promotes senescence and induces bud dormancy; stimulates blooming; and acts against certain infections [48,49,50,51,52].

### 3.2. Auxins

Auxins are plant bioregulators that significantly play important roles in plant growth facilitating the flowering of plants or plant propagation. They are also used to maintain weed-free lawns, to initiate the growth of roots in stem cuttings, and to prevent leaf and fruit fall at an early stage. They also control xylem differentiation and cell division, serve as herbicides to eradicate dicot weeds, produce fruit without fertilization, and encourage the natural detachment (abscission) of older fruits and leaves. Apical dominance can arise when the development of apical buds inhibits that of lateral buds. In such circumstances, the shot caps can be removed. Plant propagation can be performed by using hormone-treated cuttings. Also, they can increase parthenocarpy fruits and prevent pre-harvest drops of plants or sprouting by suppressing buds. Auxins suppress extended dormancy, control blooming, defoliate plants, and prevent leaf fall or abscission. Also, auxins can be used as weed killers [53,54,55,56,57].

### 3.3. Brassinosteroids

Brassinosteroids perform many functions in plants and work in conjunction with auxins for cell expansion and elongation. They participate in cell division and cell wall renewal, and initiate signal transduction that promotes vascular differentiation. They encourage pollen elongation, which is a necessary stage for pollen tube production; accelerate senescence in dying tissue culture cells; protect plants from drought stress and cold; protect plants from both biotic and abiotic stressors; and increase pesticide metabolism and removal [58,59,60,61].

### 3.4. Cytokinins

Cytokinins are involved in many aspects of plant life, like cell enlargement; cell division; morphogenesis; dormancy; apical dominance; nucleic acid metabolism; protein synthesis; and they induce flowering in short-day plants.

Their main functions include breaking bud and seed dormancy, promoting lateral bud growth, promoting cell division and apical dominance, keeping flowers fresher for longer, inducing cell division, facilitating adventitious shoot formation and lateral shoot growth, promoting nutrient mobilization, delaying leaf senescence and aging, forming new leaves and chloroplast organelles [17,62,63,64,65].

### 3.5. Ethylene

Ethylene is a naturally occurring component of ripening fruit. Ethylene is a gas that allows plants to survive at certain temperatures. Ethylene has two functions: abscission, which promotes changes related to premature abscission and the aging of leaves, petioles, flowers, and fruits; and degreening, which happens when ethylene-treated plants are exposed to air and accelerates maturity and produces uniform ripening. The most important functions of ethylene are to induce flowering, promote the sprouting of potato tubers, break the dormancy of seeds and buds, increase the respiration rate during fruit ripening, stimulate the flow of latex in rubber trees, facilitate the senescence and abscission of flowers and leaves, stimulate fruit ripening, affect horizontal growth of seedlings and swelling of the axis in dicot seedlings, and stimulate root hair development and growth, allowing the plant to extend its surface area for absorption [65,66,67,68].

### 3.6. Gibberellins

The most important functions of gibberellins consist of delaying senescence in fruits, fostering leaf expansion, fragmenting bud and seed dormancy, stimulating bolting in cabbages and beets, facilitating growth of fruits like apples and enhancing their shape, speeding up the malting process, acting as a spraying agent to increase sugarcane yield by prolonging the stem, and can be used to reduce the period of maturity and facilitate early seed production in young conifers. To boost agricultural productivity, plants like sugarcane may be grown taller, while grape stems can be grown longer. Also, gibberellins delay senescence; fruit growth; flowering; and metabolization of food in seed storage cells [20,69,70,71,72,73].

### 3.7. Jasmonates

The basic purpose of jasmonic acid and its related compounds is to control plant responses to biotic and abiotic stresses, in addition to growth and development. Growth inhibition senescence, primary root growth, tendril coiling, tuber formation, reproductive tuber formation, photosynthesis, seed germination, reproduction, flower development, and leaf abscission are all mechanisms that control plant growth. When insects attack plants, they respond by releasing jasmonates, which promote the development of protease inhibitors and other anti-herbivore defensive chemicals. Jasmonic acid has a pest control function [74,75,76].

### 3.8. Karrikins

Karrikins are a novel class of naturally occurring plant growth regulators of extensive relevance. Karrikin-specific reporter genes or other karrikin-induced phenotypes will be useful in this context. Karrikins are effective for breaking the dormancy of seeds of many species suited to areas that are frequently exposed to fire and smoke, as well as stimulating germination of the soil seed bank, the development of new plants, and responses to abiotic stress, like dehydration. Seeds from several families of blooming plants and conifers represent diverse plant forms [29,77,78].

### 3.9. Melatonin

Melatonin has both biotic and abiotic antistressant properties; increases root and leaf growth as well as being cold resistant; increases resistance to UV-B rays; increases lustrousness or biomass; reduces seed output and retards blooming; reduces the proportion of losses during postharvest storage for various fruits and vegetables; regulates root and shoot development to promote seed germination and rhizogenesis while also delaying leaf senescence; improves gene expression, enzyme activity, and photosynthesis; And protects plants from pathogens (regulates innate immunity and defensive responses) while also influencing other biochemical and physiological processes [79,80,81,82].

### 3.10. Nitric Oxide

Nitric oxide is essential for plant growth, development, and stress responses. It controls gene expression, alters phytohormones, and aids plant development and defense systems. The ideal signaling molecule is both autocrine (inside a single cell) and paracrine (between neighboring cells); causes aleurone cell vacuolation and dormancy loss; reduces the dormancy period; prevents root elongation and promotes lateral root development; encourages adventitious roots; induces the production of root hair; promotes de-etiolation; reduces hypocotyl elongation; induces stomatal closure; prevents pollen tube development; and inhibits the transition to the flowering stage.

It also promotes tracheary unit differentiation; influences the cellulose content in roots in a dose-dependent manner; enhances chlorophyll content; prevents fruit maturation; and postpones senescence [83,84].

### 3.11. Peptides

Small peptides in plants are generally less than 120 amino acids long, with physiologically active versions containing fewer than 20 amino acids. These peptides play an important role in controlling plant growth, development, and physiological activities, even at low concentrations. Peptides are utilized as antimicrobial agents, immunological stimulants, plant growth bioregulators, insecticides, or herbicides, to protect plants against pests, bacteria, weeds, and viruses. They provide an important function in long-distance signal transmission inside plants and are major communicators to a variety of stressors, including salt, alkalinity, drought, high temperatures, and cold. The are resistant to abiotic stress and play a role in long-distance transfer. The peptide systemin is responsible for the systemic defensive response in tomatoes, whereas defensins are small cysteine-rich proteins that play a role in the innate immune system of plants. CLAATA3 peptide modulates meristem growth, whereas the SCR peptide functions as a pollen self-incompatibility recognition factor. Synergid cells produce LURE peptides that attract pollen tubes to the embryo sac. RALFs are a novel family of plant peptides that promote plant cell growth [85,86,87].

### 3.12. Phenolic Compounds

The majority of phenols contribute to plant development by assisting in cell wall construction; they are also important for light energy transduction, which results in changes in plant cell wall structure, water flow, turgor pressure, and growth. Phenols are beneficial allelochemicals found in all sections of the plant, and induce nod gene expression. Phenols are necessary flavonoids that help color plant blooms and fruits, as well as pollinate and distribute seeds. They help plants defend themselves against diseases. They play an active role in the response of plants to a variety of abiotic stressors, including drought, salt, cold, and heavy metals. They affect the pools and fluxes of inorganic and organic soil nutrients and inhibit seed germination or root development. Plant-microbe interactions rely on signaling chemicals [39,88].

### 3.13. Polyamines

Polyamines play roles in several physiological processes in plants, including growth, development, abiotic stress response, defense, and aging. They play roles in a variety of plant metabolic processes, including cell division and organogenesis, embryogenesis, reproductive organ development, root growth, tuberization, floral initiation and development, fruit development and ripening, leaf senescence, seed germination, organogenesis, tissue lignification, abscission, senescence, embryogenesis, flowering, pollination, fruit development, and ripening. They respond to both biotic and abiotic stressors [89,90].

### 3.14. Strigolactones

Strigolactones play two functions in flowering plants: as hormones that govern growth and development, and as rhizosphere signaling molecules that promote symbiosis with arbuscular mycorrhizal fungi and root-parasitic weeds. They also regulate plant architecture (by inhibiting bud expansion and shoot branching), photomorphogenesis, seed germination, nodulation, and physiological responses to abiotic stimuli [45,91,92].

### 3.15. Turgorins

Turgorins play an important role in controlling physiological responses in plants. Turgorins regulate leaf mobility and nyctinasty. They are seldom mentioned in the literature as agents that perform undefined functions during temperature and damage stresses. Turgorins are a kind of signaling molecule that cause “nastic” motions in plant components in response to temperature, injury, mechanical shocks, and other stimuli that alter turgor pressure [93].

## 4. Effects of Plant Growth Bioregulators on Mammals

The interaction between the human body and the many compounds that constitute tissues and cells, also known as “chemical xenobiotics”, is especially interesting in terms of the evolutionary impact. This approach addresses the issue of the biotransformation of chemical xenobiotics introduced into the body. For this, various features of pathobiochemistry are considered, which might highlight molecular interactions between bioconstituents and chemical xenobiotics. The presence of these relationships is sometimes difficult to detect, and at other times is only revealed later through the effects of their activities over time. Chemical bioconstituent-xenobiotic interactions can result in the identification of so-called “biochemical insults”. It is thought that they constitute the first instance of xenobiotic assault. In a subsequent stage, the consequences at the cell level might be manifested/emphasized, given that “cellular injury” has happened. Furthermore, it is critical to have means to safeguard the body by limiting the amount of xenobiotics, supplying certain substances (medicines) to counteract the effects, or intervening with intrinsic factors, immunological characteristics (properties), etc. When there are no natural defense mechanisms or adequate means of intervention, the effects appear on the tissues (e.g., epithelial, connective, etc.) and organs (stomach, liver, kidneys, heart, etc.) before reaching the body, disrupting normal physiological processes. Chemical xenobiotic research focuses on the pathophysiology (pathophysiology) that impacts human health at an organismal level. The brief explanation of the evolutionary features described above might help to comprehend the necessity for preventative measures in the relationship between the environment (external/internal) and the human body, which is triggered by the interaction of bioconstituents (chemical xenobiotics). The significance of PGBRs in plants is well understood, but little research has been conducted on the effects of these natural substances on people and animals. Because PGBRs are found in all plants, whether in small or large amounts, they serve as plant-derived nourishment for numerous organisms. Once taken and ingested, the issue arises as to whether they have any effect on the organisms [94]. The effects of PGBRs on mammals are presented in the same order as PGBRs were studied. Exposure to PGBRs has been related to toxicity in several human organs, including the testis, ovaries, liver, kidneys, and brain. Furthermore, numerous PGBRs have been identified as potential endocrine disrupters. There is evidence of developmental and reproductive harm caused by prenatal and postnatal exposure in both animals and humans. PGRs can interfere with sex hormone synthesis and secretion, disrupt the structure and function of the reproductive system, and impair child growth and development, all of which may be linked to abnormal germ cell cycle, cell death, and oxidative stress [95]. Abscisic acid (ABA) was found to have immunoregulatory and anti-inflammatory effects on preclinical forms of inflammation and diabetes. ABA causes severe angiogenic disorders and includes vascular hypertrophy, exudation, cellular inflammation, and organ failure. ABA effectively inhibits endothelial tube migration, growth, and expansion while preserving cell viability. Exogenous ABA has been shown in retinal vasculature studies to impair blood vessel development and renewal. Furthermore, ABA leads macrophages to polarize toward the destructive M1 phenotype, which is defined by antiangiogenic marker expression. Similarly, ABA therapy promotes the macrophage-induced planned regression of fetal blood system [96]. ABA is a universal signaling molecule that triggers a variety of behaviors in animals via a signaling pathway that is very similar to that used by plants. This method involves the sequential binding of ABA to a membrane receptor and the activation of ADP-ribose cyclase, which results in an excess of intracellular cyclic ADP-ribose and an increase in intracellular Ca^2+^ levels. ABA induces stress responses in animal cells, immunological responses in leukocytes, and insulin release from pancreatic b cells, colon, and mesenchymal cells. ABA additionally reduces the proliferation and differentiation of cancer cells. Unlike certain medications that destroy cells, ABA acts as a growth regulator and has no notable negative effects on animal cells [97]. ABA is useful for the treatment of obesity, diabetes, atherosclerosis, and inflammatory diseases in animals. ABA lowers neuroinflammation, promotes neurogenesis, enhances synaptic plasticity, and improves memory and cognitive functions. ABA greatly improves depression, anxiety, and Alzheimer's disease. The effect of ABA on the physiological effects and therapeutic applications in related maladies was examined [98]. The human organism includes ABA from both dietary sources and endogenous generation via carotenoid biosynthetic pathways. ABA stimulates the absorption of glucose in musculoskeletal and adipose tissue using an insulin-independent pathway. Furthermore, ABA promotes the consumption of energy by both brown and white fatty tissues. ABA has a neurotrophic impact on the mammalian central nervous system and is linked to sleep, melancholy, pain, and memory [99]. ABA also helps humans maintain glucose homeostasis. In mice, ABA has been demonstrated to enhance glucose tolerance and inflammation associated with obesity. ABA and its receptor, LANCL2, play critical roles in glucose regulation and immunological modulation. Dietary low-dose ABA boosts glucose tolerance in healthy persons without boosting insulin levels, but oral ABA improves glucose tolerance in diabetic rats. The findings suggest that dietary or endogenous ABA is a distinct mammalian signaling molecule that regulates responses to environmental stresses (e.g., metabolic processes, nutritional, inflammatory, and immunologic) and that it contributes to controlling glycemic levels via an LANCL2-dependent pathway [100]. ABA enhances the activity of innate immune cells, mesenchymal and hemopoietic stem cells, and insulin-producing pancreatic cells. LANCL2 is an ABA receptor found in mammalian cells. Following N-terminal glycine myristoylation, LANCL2 is found in the plasma membrane and cytoplasmic membrane vesicles. It binds withn α subunit of a Gi protein and activates adenylate cyclase to commence the ABA signaling pathway. It was also investigated how ABA moves across the plasma membrane of human red blood cells (RBCs). ABA binds mostly to Band 3 (the RBC anion transporter). Once within the RBC, ABA stimulates ATP release by activating adenylate cyclase through the LANCL2 receptor. ATP produced by RBCs has been demonstrated to have a vasodilator effect, in which plasma ABA may play a part in regulating vascular tone [101,102].

As a reaction to stress, humans have been shown to collect autocrine ABA, as well as to use ABA to modify numerous disease-associated systems. An ABA mimetic photoaffinity probe was used on intact mammalian insulinoma and embryonic cells, which led to the identification of HSP70 family members as potential human ABA-binding proteins. An in vitro investigation of ABA-HSP70 interactions found Kds to range from 20 to 60 mM, which decreased severalfold in the presence of co-chaperone [103]. ABA has recently been demonstrated to offer powerful therapeutic advantages in a variety of clinically significant human illnesses. Human studies have firmly demonstrated that ABA retains its stress-related functional features, which were initially found in plants, resulting in improved inflammatory defense systems in mammals. Furthermore, animal studies have shown that ABA has powerful anti-inflammatory properties, as seen by a considerable reduction in immune cell infiltrates at inflammatory sites. ABA therapy eventually leads to considerable improvements in both noncommunicable and communicable diseases, which are related to a decrease in inflammation. ABA was also shown to have a wide range of physiological effects on non-immune components. One of the most prominent qualities of ABA is its capacity to activate and stimulate mesenchymal stem cells, which may open up new avenues for its use in regenerative medicine [104]. ABA can control peripheral immune response and insulin activity in rodents. Chronic ABA therapy prevents neuroinflammation produced by a high-fat diet. ABA enhanced glucose tolerance in HFD-fed rats. Chronic ABA therapy increased the cognitive performance of these animals without affecting control diet-fed mice. It also inhibited HFD-induced changes in the hypothalamus, such as the activation of microglia and TNFα production [105]. Clinical investigations have established a relationship between elevated retinoic acid (RA) and depression. ABA and retinoic acid (RA) are direct derivatives of carotenoids, with similar chemical structures. ABA also controls corticotrophin-releasing hormone (CRH) activity through the RA signaling pathway. Antidepressant activities indicate new functions for ABA in the central nervous system, perhaps leading to the development of innovative depression treatment techniques [106]. ABA is found in the brains of several animals and has neuroprotective properties. There have been several studies on the application of ABA in cognitive functioning in rats. Central ABA injections may aid in reducing muscular weakness and balance problems in rats treated with 6-hydroxydopamine (6-OHDA). The study found that ABA therapy did not significantly improve cognitive function in rats with Parkinson's disease [107]. Nanomolar ABA regulates the metabolic response to glucose availability in mammals by increasing glucose absorption in skeletal muscle and adipose tissue via an insulin-independent mechanism while lowering the consumption of energy in brown and white adipose tissues. Unlike the insulin-induced activation of AMPK-inhibiting Akt, ABA stimulation of AMP-dependent kinase (AMPK) promotes GLUT4-mediated muscle glucose uptake and has a browning effect on white adipocytes. The ingestion of micrograms per kg body weight of ABA enhances glucose tolerance in both normal and borderline subjects, and the long-term use of such a dose of ABA improves blood glucose, lipids, and morphometric parameters in borderline prediabetes and metabolic syndrome [108]. The impacts of subacute and subchronic therapy involving a couple of plant growth bioregulators (PGBRs), including abscisic acid (ABA) and gibberellic acid (GA3), on neurological and immunological markers in different rat tissues were also studied. Acetylcholinesterase (AChE) and butrylcholinesterase (BChE) were chosen as neurotoxic biomarkers, whereas ADA and MPO were evaluated as indicators of immunotoxic activity. The results show that administering PGBRs at subacute and subchronic doses raises AChE, BChE, and MPO activities while varying ADA activity in different rat tissues [109].

The effects of natural indole-3-acetic acid and indole-3-butyric acid (IAA, IBA) auxins on tumor formation and the cell cycle were studied. All drugs demonstrated cytostatic effects on certain human tumor cell lines. The cell cycle analysis shows that 2,4-D and IAA significantly lower the number of S-phase cells in the MCF-7 cell line and cause a severe G1 arrest. These data suggest that auxins have a fresh, undiscovered anticancer potential that warrants further investigation [110].

Proliferation characteristics of Indole-3-acetic acid were investigated in an in vitro model of mammalian renal tubular epithelial cells utilizing an experimental model of renal tubular epithelial cells from the LLC-PK1 cell line obtained from a healthy male pig kidney. After 72 h of adding auxin to cultivated LLC-PK1 cells, cell proliferation rose considerably as compared to the controls [111]. Indole-3-acetic acid (IAA) and chlorogenic acid (CA), two plant growth regulators, were studied for their effects on pure human and horse serum butyrylcholinesterase (BChE). IAA interacted with two enzyme species in a concentration-dependent and fast manner. Kinetic investigations revealed that IAA inhibits human serum BChE in a linear-mixed type and the horse serum enzyme in an uncompetitive manner. Human BChE has a and Ki values of 2.15 and 3.09 mM, respectively, but the Ki value of the horse enzyme is 1.05 mM [112]. IAA was also investigated for its potential adverse effects on hematological parameters, hepatorenal function, cardiac and skeletal muscles, and testes of rats, as well as histopathological changes in the aforementioned organs, as well as the extent to which any adverse effects occurred in animals after IAA withdrawal. Rats treated with IAA had anemia, leukopenia, neutrophilia, and lymphopenia, as well as significant increases in serum transaminase, gamma-glutamyl transferase, creatine kinase-myocardial band, creatine kinase-muscle type, and serum creatinine, sodium, chloride, and potassium. In addition, blood levels of testosterone, gonadotropins, and leptin were considerably reduced. After IAA removal, the bulk of the observed parameters changed. Many tissues had histological abnormalities, supporting these alterations [113]. Gibberellic acid (GA3) and indoleacetic acid (IAA) were also investigated for their impact on rat testicular function. GA3 and IAA significantly increased blood total lipids, total cholesterol, triglycerides, phospholipids, and low-density lipoprotein cholesterol, while decreasing high-density lipoprotein cholesterol, total protein, and testosterone levels. The activity of alkaline phosphatase, acid phosphatase, and gamma-glutamyl transferase all fell dramatically. There was also a substantial decrease in epididymal fructose and sperm count. At the same time, total antioxidant capacity, glutathione, sulphahydryl group concentration, superoxide dismutase, catalase, and glucose-6-phosphate dehydrogenase activity were all significantly decreased. Rats treated with GA3 and IAA showed significant testicular changes, including Leydig's cell degeneration, decreased seminiferous tubule and necrotic signals, and sperm degeneration [114]. Blood enzymes, such as lactate dehydrogenase (LDH), alkaline phosphatase (ALK-P), aspartate aminotransferase (AST), creatine kinase (CK), and serum glutamyl pyruvic transferase (SGPT), are all impacted by IAA and 2,3,5-triiodo benzoic acid (TIBA). Activity%–[I] diagrams were used to compute the inhibitory I50 values of substances. According to the findings, IAA is an activator as opposed to an inhibitor. While CK, CK-MB, amylase, ALK-P, and LDH activity rose, AST activity dropped. SGPT and GGT-P concentrations, however, were impacted by IAA. CK-MB was inhibited by TIBA and kinetin, but not by CK, amylase, ALK-P, or GGT-P. Conversely, kinetin boosted the same enzymes whereas TIBA had an impact on SGPT and LDH [115].

A sublethal dose of three PGBRs was investigated on serum enzymes in rats in a laboratory environment. For three weeks, eight rats received 100 ppm of PGBRs, IAA, indolebutiric acid (IBA), and kinetin orally at any time. Compared to the control rats, hormone treatments exhibited different effects on serum ALT, AST, LDH, amylase, and CPK levels. The results demonstrate that IBA significantly increases the levels of LDH and CPK, whereas IAA significantly increases the levels of AST, LDH, and CPK. In addition, kinetin significantly increased AST, LDH, and CPK levels. To summarize, these chemicals have toxicological effects on animals under subchronic therapy [116]. Human carbonic anhydrase I and I were tested against four widely used plant growth regulators: kinetin, gibberellic acid, indole-3-acetic acid, and indole-3-butyric acid. Sepharose-4B-L-tyrosine-1-sulfonamide affinity gels were used to purify carbonic anhydrases I and II from human erythrocytes. Activity percentage plots were used to calculate the IC50 values of the compounds that produced inhibition. The plant growth bioregulators had varying effects on human CA activity. At doses of 78.13 µM, 54.48 µM, and 62.89 µM, respectively, indole-3-acetic acid, indole-3-butyric acid, and kinetin demonstrated a 50% inhibition of hCA I. At 75.47 µM and 38.05 µM, respectively, indole-3-acetic acid and indole-3-butyric acid 50% inhibited hCA II. Gibberellic acid and kinetin, on the other hand, raised the activity of the enzyme [117].

Brassinosteroids (BRs) have received worldwide attention over the last decade due to their diverse biological actions in animal systems. A recent study demonstrated that BRs have anticancer, antiangiogenic, antiviral, and antipathogenic virus capabilities, including herpes simplex virus type 1, arenaviruses, and measles virus, as well as antigenotoxic, antifungal, and antibacterial bioactivities in animal testing. In humans, BRs inhibit viral replication and have deadly effects on malignant cell lines while not affecting normal cells. BRs significantly boost the cytotoxicity of cisplatin, a marketed antitumoral medicine, on lung cancer cells A549, reducing the IC50 value by nearly twice at low dosages. In addition to the cytotoxic and antiproliferative properties of these medicines, BR analogs have been shown to be beneficial against cutaneous psoriasis. Brassinosteroids appear to influence growth and the cell cycle via cell cycle machinery and apoptosis, making them intriguing candidates for the development of novel cancer therapies. Brassinolide effectively overcomes resistance in the human T lymphoblastoid cell line CCRF-VCR 1000 by blocking drug effusion through P-glycoprotein. Antiangiogenic capabilities of BRs may also be beneficial in inhibiting tumor growth and metastasis, opening the path for the development of various novel phytohormone-derived anticancer drugs. BRs might one day be utilized to treat cancer, as well as fungal, bacterial, and viral infections [118,119,120,121,122]. In L6 rat skeletal muscle cells (EC50 4 M), homobrassinolide (HB), a steroidal lactone with strong plant growth-promoting effects promoted protein synthesis while blocking protein degradation, which is partially mediated by the PI3K/Akt signaling pathway. In comparison to vehicle-treated controls, the oral administration of HB (20 or 60 mg/kg/day) for 24 days increased food intake, body weight gain, lean body mass, and gastrocnemius muscle mass in healthy rats fed a conventional diet [123]. The effects of HB treatment were investigated in the livers of C57BL/6J high-fat-diet-induced obese mice, concerning glucose metabolism, insulin sensitivity, body composition, and gluconeogenic gene expression patterns. Within three hours of giving obese mice an acute oral dosage of 50–300 mg/kg HB, their fasting blood glucose levels decreased in a dose-dependent manner. Without changing body weight or composition, daily chronic treatment with HB (50 mg/kg for 8 weeks) reduced hyperglycemia and enhanced oral glucose tolerance linked to obesity. Following these modifications, the liver and muscle tissues showed an increase in the phosphorylation of AMP-activated protein kinase, whereas the expression of two important gluconeogenic enzymes, phosphoenolpyruvate carboxykinase (PEPCK) and glucose-6-phosphatase (G-6-Pase), decreased [124]. Using primary keratinocyte and murine fibroblast cell cultures, proliferation and migration studies were used to investigate the biological activity of homobrassinolide (HB) in vitro. In vitro, HB slightly increased keratinocyte proliferation but enhanced fibroblast migration and proliferation.

The effects of topical HB treatment on wound closure were studied further in a mouse model of cutaneous wound healing. Mice treated with brassinosteroid medication had smaller wounds and healed faster because the inflammation and re-epithelialization were regulated during the healing process [125].

Cytokinins exhibit a variety of effects on animal cells and tissues. They protect cells from many forms of stress and reduce the harmful effects of cell aging. Cytokinins have cytoprotective, anti-aging, and antioxidant properties. Since skin fibroblasts were the first to show signs of anti-aging, several investigations have been carried out to evaluate the function of cytokinin bases in skin protection, both in vitro and in vivo. Desired outcomes include increased wound healing, protection from UV radiation, and aquaporin induction. Moreover, cytokinins control keratinocyte differentiation and melanogenesis. Numerous clinical investigations have demonstrated that they can alleviate a variety of photoaging skin characteristics in addition to specific symptoms of rosacea and acne. They also shown neuroprotective properties. It has been demonstrated that cytokinin ribosides, in addition to cytokinin bases, also exhibit neuroprotective properties. The effects of immunomodulation impact both adaptive and innate immunity. Significant cytotoxic effects have been demonstrated against a range of human cell lines generated from solid tumors and hematological malignancies by the natural cytokinin ribosides iPR, KR, BAR, ortho-topolin riboside (oTR), and N6-(2-hydroxy-3-methoxybenzyl)adenosine [126].

Cytokinin ribosides (N6-substituted adenosine derivatives) show antitumor activity both in vitro and in vivo. A comprehensive analysis was conducted to examine the correlation between the molecular makeup of cytokinins and their cytotoxic properties on a range of human cancer cell lines with different histological backgrounds. The results indicate that kinetin riboside, N6-isopentenyladenosine, and N6-benzyladenosine are cytotoxic, and that a wider variety of cell lines than previously believed are susceptible to these substances and their parent tissues. The isoprenoid cytokinin cis-zeatin riboside and the hydroxylated aromatic cytokinins ortho-, meta-, and para-topolin riboside were shown to have cytotoxic effects for the first time [127].

Kinetin was examined at low and high concentrations (1 nM–10 μM) for its physiological effects on animal cells under cytotoxic and genotoxic circumstances. Kinetin has limited antioxidant activity in the cell-free system, and high doses (500 nM or more) reduce cell viability and induce DNA damage in vitro. In contrast, small doses of kinetin (up to 100 nM) protect cells from oxidative damage. Kinetin pretreatment significantly reduces the production of reactive oxygen species mediated by 4-nitroquinoline 1-oxide. Furthermore, pretreatment with kinetin protects cellular GSH levels when combined with the GSH-depleting medication patulin. The results unequivocally demonstrate that kinetin, even at low levels, inhibits apoptosis and shields cells from oxidative stress-induced cell death [128]. Natural cytokinins were examined for their effects on glutamate (Glu)-induced death and salsolinol (SAL)-induced toxicity in neuron-like dopaminergic SH-SY5Y cells, a model of Parkinson’s disease. In the Parkinson’s disease model produced by SAL, kinetin-3-glucoside, cis-zeatin riboside, and N6-isopentenyladenosine all showed action. Trans-, cis-zeatin, kinetin, iron chelator deferoxamine (DFO), and necroptosis inhibitor necrostatin 1 (NEC-1) all markedly decreased the rate of cell death in the Glu-induced model. Studies using lactate dehydrogenase showed that DFO and NEC-1 had a greater neuroprotective impact on neurons than cytokinins. Moreover, they had a reduced effect on apoptotic caspase-3/7 activity reduction than DFO. The cytokinins produced superoxide radicals in a manner akin to those of DFO and the NEC-SAL-induced model of Parkinson’s neuronal cell death, and the Glu-induced model of oxidative damage generally showed protective action, mostly through the reduction in oxidative stress [129].

Gibberellin was used to measure human sperm motility. In vitro, samples treated with varied quantities of gibberellin decreased spermatozoa motility. In addition, exposure to gibberellin suppresses the activities of Na^+^/K^+^-adenosine triphosphatase (ATPase) and Ca^2+^-ATPase, which preserve ion stability both inside and outside spermatozoa membranes and increases the amounts of reactive oxygen species and apoptotic marker protein in human sperm. Additionally, the treatment of gibberellin decreased the levels of adenosine triphosphate synthase and adenosine triphosphate synthesis, which may have encouraged the phosphorylated form of adenosine 5′-monophosphate-activated protein kinase (AMPK) and its protein synthesis. Gibberellin decreases ATPase function and raises reactive oxygen species levels in vitro, which decreases human sperm motility. This may cause AMPK to be overexpressed, which would decrease the ability of spermatozoa to fertilize [130]. The histological and histochemical effects of Gibberellin A3 (GA3) on the livers of albino rats were investigated. Numerous histological alterations in the liver were caused by 24 p.p.m. of GA3 administered by gavage in 0.2 mL saline three times a week for three weeks. These included inflammatory leucocytic infiltrations, blood vessel congestion, and cytoplasmic vacuolization of hepatocytes with pyknotic nuclei. Total protein and glucose concentrations of hepatocytes significantly decreased, according to the histochemical analysis. Serum levels of the enzymes GOT, GPT, and alkaline phosphatase were considerably lower, especially after the third week [131]. Given that the kidney is one of the primary target organs for many different toxins, a study of the potential harmful effects of plant growth regulators on this organ is particularly interesting, given their widespread usage in agriculture. To ascertain subacute toxicity and subchronic damage, researchers examined the effects of gibberellic acid on the renal cortex of adult male albino rats for two weeks and two months.

Measurements of serum urea and creatinine revealed that the treated subacute and subchronic groups had higher levels than the control group. These indicators suggested that cell membrane integrity and cellular leakage of kidneys were both present [132]. Long-term GA3 ingestion accelerated oxidative damage and tumor development. Substance P (SP) and mast cells both contribute significantly to inflammation. For 30 days, Wistar albino rats received either one or more GA3 doses. Mast cell recruitment and activation in both tissues were markedly enhanced by subchronic GA3 treatment. SP levels in the skin and bladder reduced following a 30-day course of therapy with 2 mg/kg GA. The bladder’s SP levels recovered to normal and the skin’s rose following a 30-day course of therapy with 20 mg/kg GASingle GA3 dosages reduced SP levels in the skin while increasing mast cell recruitment and activation. Our findings suggest that exposure to plant growth regulators may worsen inflammatory skin conditions since both SP and mast cell activation promote inflammatory responses [133].

Both in vitro and in vivo, jasmonates have anti-cancer properties. In addition to extending the life span of EL-4 lymphoma-bearing mice, jasmonates have been shown to selectively kill cancer cells while sparing normal blood lymphocytes—even when those cells were a component of a mixed population of leukemic and normal cells extracted from the blood of patients with chronic lymphocytic leukemia (CLL). A plethora of novel and old plant-derived cancer chemotherapeutic medications are being developed, including jasmonates. The activities of jasmonates on plant and cancer cells have been discovered to have several similarities. This suggests that more research into the effects of jasmonates on plant cells may help to clarify the anti-cancer properties of these substances. The activation of MAPK, creation of ROS, promotion of cell death, inhibition of proliferation and cell cycle arrest, and increased expression of heat-shock protein (HSP) are among the similarities [134]. Plant responses to wounds and pathogens are regulated by methyl jasmonate (MJ). Additional studies used animal pain models to examine the antinociceptive qualities of MJ. The acetic acid-induced writhing, tail immersion, formalin-induced paw licking, and Randall-Selitto paw pressure tests were used to evaluate the antinociceptive effect of MJ (10–50 mg/kg) administered intraperitoneally (i.p.) to mice. Mice’s abdominal constriction brought on by acetic acid was avoided by MJ. Additionally, it significantly reduced the inflammatory discomfort that the second part of the formalin test in mice produced. In the first half of the formalin test, MJ had no effect on neurogenic pain; in the tail immersion test, MJ did not influence the mice’s response time to excruciating heat. In rats, MJ had no impact on pain sensitivity in the noninflamed hind paw, but dramatically increased paw withdrawal latency in the inflamed hind paw in the Randall-Selitto paw pressure test.

At doses ranging from 100 to 300 mg/kg, the acute toxicity test on mice showed that MJ administered intraperitoneally was well tolerated and did not cause any toxic symptoms or fatalities [135]. When plants are exposed to stress, their response and signaling pathways are influenced by strigolactones (SLs), which are produced from carotenoids. They also affect a multitude of mammalian cellular functions and provide novel scaffolding for an array of biological applications. Hyperglycemia and type 2 diabetes are characterized by insulin resistance. Carbon dioxide levels and energy metabolism are regulated by SIRT1, an NAD+-dependent deacetylase. The sensitivity of skeletal muscle tissue to insulin is enhanced by SIRT1 activators, particularly plant polyphenols, like resveratrol. In skeletal muscle cells, strigolactone and pinosylvin, which are produced by plants, improve SIRT1 activity, insulin signaling, glucose uptake, and mitochondrial biogenesis. Treatment of the L6 skeletal muscle myotubes of rats with pinosylvin and strigolactone analog GR24 was conducted. Insulin signaling, glucose uptake, GLUT4 translocation, and mitochondrial biogenesis were all enhanced by strigolactone GR24’s enhancement of SIRT1 activity without AMPK activation. Novel therapeutic methods for the treatment of insulin resistance in skeletal muscle. may be provided by the control of SIRT1 by strigolactone GR24 and the activation of AMPK by pinosylvin [136]. Studies on a variety of cancer cells have revealed that SLs significantly reduce the growth of cancer. The growth and survival of human brain tumor cells were seen to be inhibited by SL analogs. Inducing apoptosis, stopping the G1 cell cycle, and inhibiting the growth of cancer cells are all possible at low concentrations of two important bioactiphores: indanone-derived SL and EGO The genes *Bax/Caspase-3* and *Bcl-2* were expressed more highly in SL analogs than in *Bcl-2* types [137]. The use of strigolactone in recent research was the subject of two thorough reviews. Strigolactones can decrease angiogenesis, potentially boosting their anticancer activities. Additionally, it has been discovered that strigolactones are antioxidants and anti-inflammatory. The strigolactone analog may be effective against human ailments due to its antibacterial and antiviral properties, according to a few studies. Furthermore, the effects on human cells and potential medical uses of SLs have been clarified by several studies conducted in recent years. For instance, it has been shown that SLs are crucial for regulating inflammatory and apoptotic processes. More research into their effects on human cells and possible use as antibacterial and anticancer medications has been suggested by our growing understanding of the molecular processes underlying their activity [138,139].

## 5. Concluding Remarks

In this review, we presented a comparison of the activity on plants and the effect on mammals of plant growth bioregulators. Plant growth bioregulators have some common features: they are synthesized by plants, cause metabolic changes, their effects are present even at very low concentrations, and some of them can affect mammalians. Both, activity in correlation with structure and the effect on mammals are very diverse. The positive effects of these compounds were mainly highlighted.

## Data Availability

The original contributions presented in this study are included in this article; further inquiries can be directed to the corresponding author.
